# Association Between Diagnostic Delay and Short-Term Outcomes in Patients with Radiographic Axial Spondyloarthritis: Results from the Regisponser-AS Registry

**DOI:** 10.3390/jcm14061977

**Published:** 2025-03-14

**Authors:** María Lourdes Ladehesa-Pineda, Desirée Ruiz-Vilchez, Antonio Manuel Barranco, María Ángeles Puche-Larrubia, Pilar Font-Ugalde, Raquel Ena María Granados, Jordi Gratacós-Mastmijà, Xavier Juanola, Alejandro Escudero-Contreras, Eduardo Collantes-Estévez, Clementina López-Medina

**Affiliations:** 1 Rheumatology Department, Reina Sofia University Hospital, 14004 Cordoba, Spain; mangeles.puche@gmail.com (M.Á.P.-L.); raquegranados@gmail.com (R.E.M.G.); alexcudero2@gmail.com (A.E.-C.); h72lomee@uco.es (C.L.-M.); 2 Maimonides Biomedical Research Institute of Cordoba (IMIBIC), 14004 Cordoba, Spain; desiree.ruiz@imibic.org (D.R.-V.); anto.13.moyano@gmail.com (A.M.B.); fougp@hotmail.com (P.F.-U.); educollantes@yahoo.es (E.C.-E.); 3 Medical and Surgical Department, University of Cordoba, 14004 Cordoba, Spain; 4 Rheumatology Department, University Hospital Parc Tauli, 08208 Sabadell, Spain; jgratacosmas@gmail.com; 5 Universitat Autonoma de Barcelona, 08193 Barcelona, Spain; 6 Rheumatology Department, Bellvitge University Hospital, L’Hospitalet de Llobregat, 08907 Barcelona, Spain; x.juanola@gmail.com

**Keywords:** axial spondyloarthritis, structural damage, diagnostic delay

## Abstract

**Objectives**: To evaluate whether the diagnostic delay in patients with radiographic axial spondyloarthritis (r-axSpA) is associated with poorer short-term outcomes after two years of follow-up. **Methods**: This was an observational, longitudinal, and prospective study including patients with r-axSpA from the national multicentre Spanish REGISPONSER-AS registry. Patients were divided into two groups according to the mean diagnostic delay (<5 years, ≥5 years). Binary logistic regression models adjusted for disease duration were constructed and used to evaluate the association between diagnostic delay and disease outcomes at two years. The retention rate for first-line treatment with anti-TNF across the groups was evaluated using a log-rank test. **Results:** A total of 565 patents were included. The mean diagnostic delay was 5.6 ± 6.2 years, with 325 patients experiencing a delay of <5 years and 240 patients experiencing a delay of ≥5 years. A diagnostic delay of ≥5 years was associated, after 2 years, with a higher prevalence of inflammatory bowel disease (IBD) (OR 2.01 (95%CI 1.06–3.83)), a lower prevalence of synovitis (OR 0.68 (95%CI 0.47–0.98)) and dactylitis (OR 0.24 (95%CI 0.11–0.55)), and worse disease activity after adjusting by disease duration. However, no impact was observed on quality of life, structural damage, or work disability, probably due to the short follow-up period. Finally, no differences between the groups were found with regard to the retention rate for first-line anti-TNF treatment. **Conclusions**: Diagnostic delay is associated with poorer short-term outcomes in terms of structural damage, dactylitis, and disability in patients with r-axSpA.

## 1. Introduction

The most recent classification criteria of spondyloarthritis (SpA) allow us to distinguish axial and peripheral phenotypes according to the patient’s predominant symptoms [1]. Axial SpA (axSpA) is characterised by inflammation of the spine that causes chronic inflammatory back pain and stiffness and may lead to structural damage determined by new bone formation (resulting in the appearance of syndesmophytes and bone bridges), potentially leading to irreversible ankylosis of the spine and sacroiliac joints (SIJs) [2]. In the spine, ossification of the anterior vertebral ligament is observed by radiography. This type of damage is evaluated using radiographic scoring systems such as the modified Stoke Ankylosing Spondylitis Spinal Severity Score (mSASSS) [3] and the Bath Ankylosing Spondylitis Radiology Index (BASRI) [4]. In addition, the modified New York criteria (mNY) [5] for SIJ radiographic assessment have traditionally been the basis of axSpA diagnosis, but inflammation often begins many years before radiographic signs can be observed on radiographs, and the diagnosis of radiographic axSpA (r-axSpA), previously known as ankylosing spondylitis (AS), becomes possible. On the other hand, the introduction of magnetic resonance imaging (MRI) of the SIJ has allowed earlier diagnosis of the disease [6].

As a result of structural damage to the spine, patients suffer limitations in their mobility, and due to pain, fatigue, and impaired physical function, they may also experience considerable physical, economic, and emotional burdens that affect their quality of life and mental health [7]. Moreover, persistence of inflammation and increased structural damage have been associated with comorbidities in patients with axSpA, especially osteoporosis [8] and cardiovascular disease [9]. Additionally, axSpA frequently affects peripheral joints and entheses and is associated with extra-musculoskeletal manifestations (EMMs), including uveitis, psoriasis, and inflammatory bowel disease (IBD) [10].

Therefore, early diagnosis and treatment before irreversible changes occur are crucial for managing patients with axSpA, as they can help prevent structural damage, reduce disease progression, and improve long-term functional outcomes and quality of life. Nevertheless, diagnostic delay, which can be defined as “the time from onset of first symptoms to getting a diagnosis”, is typically longer in patients with axSpA than in patients with other rheumatic diseases [11]. Recent studies report a mean diagnostic delay of between 2 years and 6 years, with variations among countries [12], in patients with axSpA, and this value has decreased only marginally over the last decades. Diagnostic delay in axSpA is associated with socioeconomic factors, lower educational attainment, the absence of extra-articular manifestations, disease presentation, HLA-B27 negativity, female sex, psoriasis, and younger age at onset [13,14]. The impact and long-term consequences of diagnostic delay have been previously assessed and appear to be related to irreversible damage in terms of quality of life and functional impairment [15]. However, the impact of the delay in diagnosis on the short-term results in patients with r-axSpA, especially on the structural damage objectifiable in radiographic and/or clinical terms, including a functional assessment within a period of less than two years, has not been evaluated.

The aims of this study were to assess whether delayed diagnosis in patients with r-axSpA is associated with worse short-term outcomes after two years and to analyse the retention rate for first-line treatment with anti-TNF antibody according to diagnosis delay.

## 2. Materials and Methods

### 2.1. Study Design and Patients

This study was a longitudinal, observational, prospective 2-year follow-up study that included 729 patients from the national multicentre Spanish REGISPONSER-AS registry. This registry is a subdivision of the main Spondyloarthritis Registry of the Spanish Rheumatology (REGISPONSER), developed by the Spanish Group for the Study of Spondyloarthritis (GRESSER) [16]. A total of 21 centres participated in this multicentre study. The REGISPONSER-AS registry includes only patients meeting the modified New York criteria for AS [5], with a diagnosis confirmed by a rheumatologist. The methodology and data collection procedures for the REGISPONSER and REGISPONSER-AS registries have been previously described [16,17]. Briefly, to be included, patients had to be adults with a confirmed diagnosis of AS, meet the New York criteria, have blood tests available within 15 days of the visit, and undergo a complete radiographic evaluation within the previous year. Additionally, patients were required to complete self-administered questionnaires. At enrolment, patients ranged in age from 20 to 81 years. The total follow-up period for the patients in these registries was 5 years, with one visit per year, although, in this study, we considered only the first two years.

All patients whose data are included consented to participate in the REGISPONSER-AS registry, which was approved centrally by the Ethics Committee of the Reina Sofia University Hospital in Cordoba (Spain).

### 2.2. Collected Data

A case report form was used to collect all data. The following variables were considered in the present study:

- Sociodemographic data: Sex, age, and smoking status.
- Clinical characteristics and SpA features: Age at onset of SpA, disease duration (years between SpA diagnosis and the study visit) [18], diagnostic delay (years between symptom onset and SpA diagnosis), family history of SpA, HLA-B27 antigen status, C-reactive protein level (CRP, mg/dL), synovitis, psoriasis, inflammatory bowel disease (IBD), enthesitis, dactylitis, uveitis, swollen joints, and hip replacement.
- Patient-reported outcomes (PROs): Disease activity was assessed using the Bath Ankylosing Spondylitis Disease Activity Index (BASDAI) [19], the patient-reported global visual analogue scale (global VAS), and the Ankylosing Spondylitis Disease Activity Score (ASDAS) [20]. Functional status was evaluated with the Bath Ankylosing Spondylitis Functional Index (BASFI) [21]. Structural damage was assessed using the Bath Ankylosing Spondylitis Radiology Index (BASRI), as it was the available tool at the time of data collection [4]. Additionally, the participants completed the Mental Health Survey (MSF12) and the Physical Health Survey (FSF12) to evaluate health-related quality of life [22].
- Past and current treatment: Data on previous or concurrent treatments were collected, including nonsteroidal anti-inflammatory drugs (NSAIDs), conventional synthetic disease-modifying antirheumatic drugs (csDMARDs) (sulfasalazine, methotrexate, or leflunomide) and biologic DMARDs (bDMARDs) (anti-TNF treatment). The dates of bDMARD initiation and withdrawal were also collected.

### 2.3. Statistical Analysis

For this analysis, we selected patients with available data for the diagnosis delay variable, as well as with a limit of disease duration of 30 years. The exclusion of patients with a disease duration of more than 30 years was based on three main reasons: (a) the very low frequency of extreme values; (b) improved model fit and data distribution; and (c) clinical plausibility, as prolonged disease duration can influence several outcomes. The patients were divided into two groups according to their mean diagnostic delay (<5 years, ≥5 years), since the use of median values is advisable when analysing skewed data [11]. This cut-off of 5 years coincides with the median diagnostic delay in the overall REGISPONSER population.

Descriptive data are presented herein as the mean and standard deviation (SD) for continuous variables and as absolute frequencies and percentages for categorical variables. Comparisons between diagnostic delay <5 years and ≥5 years were made using the χ2 test or Fisher’s exact test for categorical variables and Student’s independent T test for continuous variables. Binary logistic regression models adjusted for disease duration were constructed and used to evaluate the association between diagnostic delay and disease outcomes at two years. This variable was considered a confounding factor since it may be associated with both the clinical characteristic and the diagnostic delay.

Finally, the retention rate after two years for first-line treatment with anti-TNF antibody was compared between both groups using a log-rank test. Data were censored at two years, meaning that patients who remained on treatment beyond this period were considered censored (i.e., no event occurred).

All contrasts were bilateral and considered significant at a *p*-value <0.05. Data were processed and analysed using SPSS version 25.

### 2.4. Handling of Missing Data

Patients for whom data on the date of diagnosis and time of onset of first symptoms were available were included in the study. If an outcome at two years was missing, it was omitted, and the remaining data were analysed (listwise deletion).

## 3. Results

### 3.1. Description of the Population

Among the total of 729 r-axSpA patients belonging to the REGISPONSER-AS cohort, 565 were included in the analysis. The mean diagnosis delay was 5.6 ± 6.2, with 325 and 240 having a diagnostic delay <5 and ≥5 years, respectively. Most patients were male (72.2%) and the mean age was 44.9 ± 10.9 years, with the <5-year delay group being older compared to those with a ≥5-year diagnostic delay. The overall population had a mean disease duration of 16.0 ± 8.2 years, with no significant differences between groups. The prevalence of HLA-B27 positivity was 436 (80.1%). At the time of inclusion in the study, the mean BASRI score, used to assess radiographic damage, was 6.5 ± 3.6, with similar values between groups. The prevalence of patients who had ever received anti-TNF treatment was 30.7% (105 patients), with no significant differences between diagnostic delay groups. Additional baseline characteristics of the patients in each diagnostic delay group at the time of study inclusion are presented in Table 1.

### 3.2. Impact of Diagnostic Delay After Two Years

In terms of clinical characteristics, patients with a longer diagnostic delay showed a lower frequency of dactylitis (OR 0.24 (95%CI 0.11–0.55)) and synovitis (OR 0.68 (95%CI 0.47–0.98)) after adjusting the model for disease duration (Table 2). Conversely, the prevalence of IBD was higher in patients with ≥5 years of diagnostic delay compared to those with <5 years (OR 2.01 (95%CI 1.06–3.83)). No significant differences were found for psoriasis or uveitis (Table 2).

**Figure 1:**
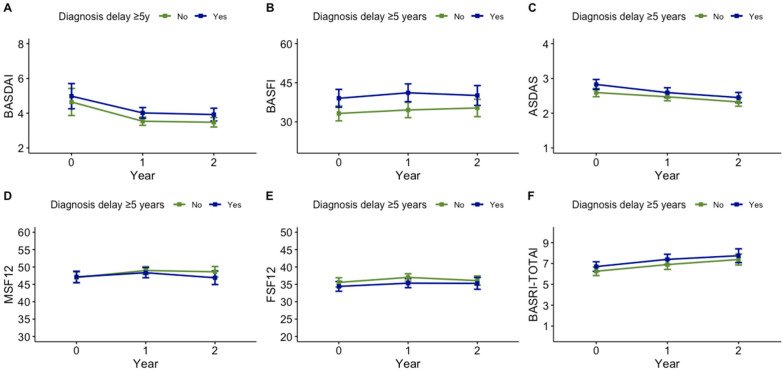
Impact of diagnosis delay on PROs, composite outcomes, and structural damage over time. The subfigures (**A**–**F**) represent the mean trend of each outcome over time. ASDAS: Ankylosing Spondylitis Disease Activity Score; BASDAI: Bath Ankylosing Spondylitis Disease Activity Index; BASRI: Bath Ankylosing Spondylitis Radiology Index.

Regarding disease activity, the mean BASDAI was higher in patients with ≥5years of diagnostic delay after 2 years of follow up, even after adjusting for disease duration (Table 3 and Figure 1). Achieving a BASDAI < 4 was less frequent among patients with a longer diagnostic delay (64.2% vs. 72.5%); however, these differences disappeared after adjusting for disease duration.

**Figure 2:**
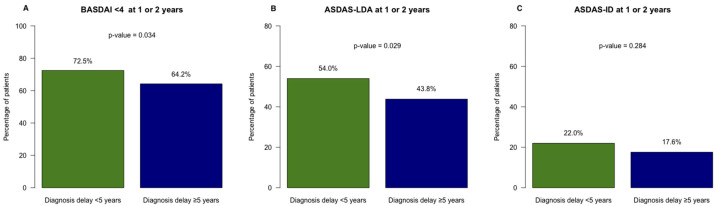
Impact of diagnosis delay on BASDAI and ASDAS after 2 years. Prevalence of patients who achieved each outcome after 1 or 2 years. (**A**) BASDAI: Bath Ankylosing Disease Activity Index; (**B**) ASDAS LDA: ASDAS low disease activity (ASDAS < 2.1); (**C**) ASDAS: Ankylosing Spondylitis Disease Activity Score; ASDAS-ID: ASDAS inactive disease (ASDAS < 1.3).

Moreover, achieving ASDAS-LDA at 1 or 2 years was less likely in patients with ≥5 years of diagnostic delay compared to those with <5 years (43.8% vs. 54.0%) (Table 3 and Figure 2). Interestingly, these differences persisted after adjusting for disease duration (OR 0.66 (95%CI 0.46–0.94)) (Table 3). No significant differences were observed between groups regarding BASFI, SF12, BASRI, mobility, or work disability after 2 years.

**Figure 3:**
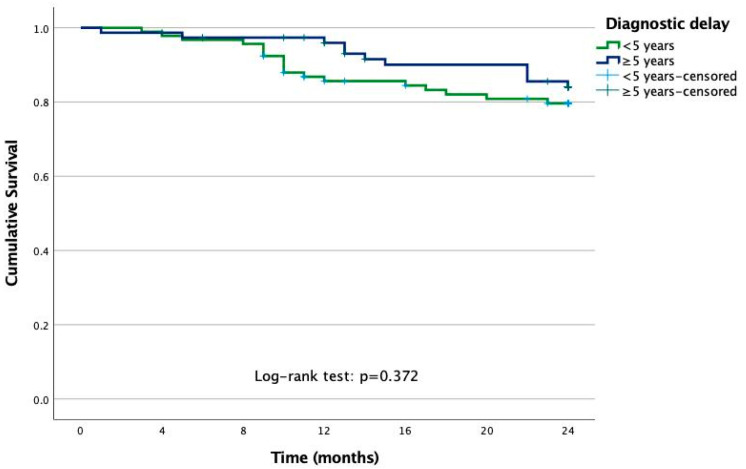
Impact of diagnosis delay on the adherence to the fist anti-TNF treatment: Kaplan–Meier curve and Log-rank test.

The survival analysis assessing whether diagnostic delay was associated with a lower retention rate for first-line anti-TNF treatment showed no significant differences between the groups (log-rank test: *p* = 0.372) (Figure 3). Patients with a diagnostic delay of ≥5 years had a mean time on anti-TNF treatment of 22.6 months (95% CI: 21.5 to 23.6), while those with a diagnostic delay of <5 years had a mean survival time of 21.5 months (95% CI: 20.3 to 22.6). Median survival could not be calculated, as neither group had more than 50% withdrawals.

## 4. Discussion

This study was performed to assess whether diagnostic delay is associated with worse short-term outcomes in terms of structural damage and disability, based on a large nationwide population in Spain. In this registry, a diagnostic delay of five or more years was associated, after two years of follow-up, with a higher prevalence of IBD, a lower prevalence of synovitis and dactylitis, and worse disease activity adjusting by disease duration. However, no impact was observed on quality of life, structural damage, or work disability, probably due to the short follow-up period.

Previously, many studies have been performed to identify factors associated with diagnostic delay in SpA [15]; however, few have analysed its impact, and most of them reported long-term outcomes regarding radiographic damage, BASDAI and BASFI scores, and spine mobility [23]. To our knowledge, this is the first study to report worse outcomes over a short period such as two years.

In the Spanish REGISPONSER-AS database, created nineteen years ago, the mean diagnostic delay was 8.0 (9.5) years. However, in our specific analysis, we restricted the population to patients with less than 30 years of disease duration, which explains why the diagnostic delay in our analysis is slightly lower. The diagnostic delay in the RESGIPONSER-AS registry is similar to the one reported by Merino M et al. in Spanish r-axSpA patients in a study about the burden of the disease in Spain, in which they described a mean diagnostic delay of 8.4 ± 7.6 years [24]. The time reported in our study is longer than that reported in a French study by Behar et al. (2017), which found a delay of 4.9 ± 6.3 years with a median of 2.0 years [25]. Furthermore, a meta-analysis by Zhao et al. (2021) found a pooled worldwide mean diagnostic delay of 6.7 years (95% CI 6.2, 7.2) with high heterogeneity [13]. Because of the high heterogeneity reported in that study, we decided to distinguish two groups of axSpA patients in our study according to the mean diagnostic delay since we believe this is a more precise parameter. The mean diagnostic delay of five years found in the REGISPONSER-AS registry in our study is consistent with previous studies performed using other national registries, such as in the UK [11].

Worse outcomes regarding disease activity, function, spinal mobility, and/or radiographic damage [26] were previously reported by Seo et al. (2015) to be related to diagnostic delay, but those authors did not specify when the outcomes were measured. Similarly, in patients with psoriatic arthritis, worse outcomes in terms of structural damage (including the presence of erosions) and quality of life have been reported for patients with diagnostic delays longer than 6 months [27]. Evidence shows that prolonged diagnostic delay is associated with poorer long-term outcomes, including worse functional impairment, greater incidence of radiographic progression, poorer quality of life, and reduced response to treatment [15]. Prolonged diagnostic delay also leads to greater work disability, unemployment, and increased health care costs [13,28]. In our study, we did not find a significant impact on quality of life or work disability, likely because a two-year follow-up was not sufficient to detect differences in outcomes that are typically associated with long-term follow-up. This is in line with the recent findings published by Berbel-Arcobé et al. [29], who demonstrated that diagnostic delay was associated with increased health costs but not with burden after a 3-year follow-up. Conversely, a meta-analysis suggested that diagnostic delay was associated with worse quality of life [30]. However, as the mean diagnostic delay in that study ranged between 4 and 12 years, this effect may be influenced by both the duration of follow-up and the length of the diagnostic delay. Further studies comparing short-term and long-term follow-ups could be useful in better understanding these implications.

From an intestinal point of view, gut dysbiosis has been postulated as an early step in the pathogenesis of axSpA [31,32], and current investigations have demonstrated subclinical inflammation of the gut in patients with axSpA [33,34]. Therefore, delayed diagnosis and, thus, treatment establishment in patients with axSpA could favour the development of signs and symptoms of IBD in these patients. Additionally, due to the nonspecific symptoms of IBD in some patients, the absence of an axSpA diagnosis and rheumatologist follow-up can cause delayed referral to a gastroenterologist. Our results indeed showed a higher prevalence of IBD among patients with a longer diagnostic delay. A recent study published study by Michelena X et al. found that a longer diagnostic delay was associated with a higher probability of uveitis and IBD in r-axSpA, suggesting that a longer period of uncontrolled inflammation might influence the incidence of EMMs. In a multivariable Cox model, r-axSpA patients with a longer diagnostic delay (≥5 years) (same median as in our study) had more IBD incidence (HR 1.85; 95% CI: 1.28, 2.67) when adjusted for age of onset, sex, and location [35]. Interestingly, we found a lower prevalence of dactylitis and synovitis among patients with a longer diagnostic delay. This could be explained by the fact that the presence of these peripheral manifestations may facilitate diagnosis and reduce the diagnostic delay in axSpA patients.

Regarding the impact of diagnosis delay on the retention of anti-TNF treatment, no association was found with the retention rate for treatment with first-line anti-TNF agents in our registry. Another study reported that BASDAI scores were significantly worse in a group of patients with late diagnosis than in a group with earlier diagnosis [26], and it appeared that r-axSpA patients with a shorter disease duration were more likely to respond to biologic agents than were patients with a long-standing disease duration [36]. Therefore, earlier diagnosis could improve the response to treatment and delay the onset of structural damage and impaired function and mobility in axSpA patients.

Important strengths of this study are its use of a large nationwide sample of patients with r-axSpA and its prospective design; these characteristics contribute to the reliability of the results. Another strength is the well-balanced treatment strategies between groups, as both populations had a similar use of anti-TNFs and csDMARDs.

Diagnostic delay in axSpA is still a challenge, and health care providers should attempt to develop various strategies for improving it so that proper treatment can be initiated earlier, and irreversible chronic damage and disability can be avoided in these patients. Education on axSpA among general practitioners, increased awareness about the early musculoskeletal and extra-musculoskeletal manifestations, and the implementation of referral pathways across specialties could help reduce diagnostic delay. In this context, increasing efforts are being made toward the development and validation of diagnostic tools that integrate biomarkers, demographic factors, clinical parameters, and imaging data, promoting earlier and more accurate diagnoses of axSpA.

One limitation is that the present results are based on an observational cohort, whereas a clinical trial assessing the impact of symptom duration on disease outcomes could provide stronger information. Another limitation is the exclusive use of BASRI to evaluate the structural damage, while modern imaging methods, such as MRI or MRI-based synthetic CT [37], could provide additional information. However, BASRI has been demonstrated to be a simple and feasible tool for structural damage assessment.

## 5. Conclusions

In conclusion, this study showed that a diagnostic delay of five or more years was associated, after two years of follow-up, with a higher prevalence of IBD, a lower prevalence of synovitis and dactylitis, and worse disease activity. However, no impact was observed on quality of life, structural damage, or work disability, likely due to the short follow-up period. Increasing efforts to reduce the diagnostic delay in these patients is crucial to improve long-term outcomes.

## Figures and Tables

**Table 1:** Description of the population at baseline.

	Total N = 565	Diagnostic Delay < 5 Years N = 325	Diagnostic Delay ≥ 5 Years N = 240	*p*-Value *
Age	44.9 (10.9)	45.2 (12.4)	41.8 (10.6)	0.022
Sex (male)	157 (27.8%)	241 (74.2%)	167 (69.6%)	0.231
Age at diagnosis (years)	34.4 (10.9)	33.0 (11.9)	31.5 (10.4)	0.188
Disease duration (years)	16 (8.2)	12.2 (8.0)	11.3 (7.6)	0.210
HLA-B27 positive	436 (80.1%)	248 (80.5%)	188 (79.7%)	0.804
Family history of SpA	310 (54.9%)	177 (54.5%)	133 (55.4%)	0.563
Psoriasis	56 (9.9%)	35 (10.8%)	21 (8.8%)	0.413
Inflammatory bowel disease	38 (6.7%)	18 (5.5%)	20 (8.3%)	0.190
Uveitis	115 (20.5%)	58 (18.0%)	57 (23.8%)	0.087
Enthesitis	176 (31.6%)	104 (32.3%)	72 (30.6%)	0.677
Dactylitis	41 (7.3%)	30 (9.3%)	11 (4.6%)	0.036
Synovitis	181 (32.1%)	113 (34.8%)	68 (28.5%)	0.112
ASDAS-CRP	2.7 (1.1)	2.5 (1.0)	2.4 (1.0)	0.321
BASDAI	4.2 (2.4)	3.7 (2.4)	3.5 (2.2)	0.281
BASFI	35.8 (26.6)	32.7 (26.7)	27.8 (23.8)	0.103
Total BASRI	6.5 (3.6)	6.3 (3.7)	5.8 (3.6)	0.213
csDMARDs ever	154/345 (44.6%)	90/196 (45.9%)	64/149 (43.0%)	0.583
Anti-TNF ever	105/342 (30.7%)	58/195 (29.7%)	47/147 (32.0%)	0.658
Inability to work	110 (19.7%)	69 (17.2%)	41 (13.4%)	0.426

Data are shown as the mean (standard deviation) or frequency (percentage). * The chi-square test and the *t* test were used to analyse qualitative and quantitative variables, respectively.

**Table 2:** Clinical characteristics at 2 years adjusted by disease duration.

	Diagnostic Delay < 5 Years N = 325	Diagnostic Delay ≥ 5 Years N = 240	OR (95%CI) Adjusted for Disease Duration
Psoriasis	37 (11.7%)	23 (9.9%)	0.83 (0.48–1.45)
Inflammatory bowel disease	19 (6.0%)	23 (10.0%)	2.01 (1.06–3.83)
Uveitis	72 (22.9%)	52 (22.4%)	0.92 (0.61–1.39)
Enthesitis	53 (18.3%)	48 (21.4%)	1.17 (0.76–1.82)
Dactylitis	36 (11.7%)	8 (3.4%)	0.24 (0.11–0.55)
Synovitis	114 (36.2%)	66 (28.8%)	0.68 (0.47–0.98)

CI: confidence interval; OR: odds ratio.

**Table 3:** Disease activity, patient-reported outcomes (PROs), structural damage, and clinimetry at 2 years adjusted by disease duration.

	Diagnostic Delay < 5 Years N = 325	Diagnostic Delay ≥ 5 Years N = 240	OR (95%CI) Adjusted for Disease Duration
ASDAS-CRP, mean (SD)	2.6 (1.1)	2.3 (0.9)	1.09 (0.92–1.30)
BASDAI, mean (SD)	3.6 (2.2)	3.3 (2.1)	1.08 (1.00–1.16)
BASFI, mean (SD)	33.8 (26.8)	30.3 (25.0)	1.01 (0.99–1.01)
Physician VAS (cm)	3.0 (1.9)	2.8 (2.2)	1.07 (0.98–1.16)
Nocturn VAS (cm)	3.6 (2.8)	3.2 (2.4)	1.03 (0.97–1.09)
Global VAS (cm)	4.0 (2.7)	3.6 (2.4)	1.05 (0.98–1.11)
Pain VAS (cm)	3.9 (2.7)	3.4 (2.4)	1.03 (0.96–1.09)
SF-12 physical component	2.4 (0.7)	2.3 (0.8)	0.87 (0.69–1.09)
SF-12 mental component	3.5 (0.7)	3.5 (0.9)	0.96 (0.77–1.20)
ASDAS LDA	113 (37.7%)	71 (32.7%)	0.87 (0.60–1.27)
ASDAS ID	44 (14.7%)	25 (11.5%)	0.83 (0.49–1.42)
ASDAS LDA at 1 or 2 years	161 (54.0%)	91 (43.8%)	0.66 (0.46–0.94)
ASDAS ID at 1 or 2 years	64 (22.0%)	36 (17.6%)	0.76 (0.48–1.19)
BASDAI < 4	200 (61.7%)	128 (53.3%)	0.75 (0.53–1.06)
BASDAI < 4 at 1 or 2 years	235 (72.5%)	154 (64.2%)	0.74 (0.51–1.07)
Spine BASRI	6.4 (3.3)	5.8 (3.3)	1.01 (0.94–1.08)
Total BASRI	7.1 (3.8)	6.3 (3.5)	1.00 (0.94–1.06)
Schober (cm)	3.2 (1.5)	3.8 (1.7)	0.96 (0.87–1.07)
Chest expansion (cm)	4.3 (2.4)	4.5 (2.0)	0.95 (0.87–1.03)
Distance to the ground (cm)	15.5 (13.7)	15.6 (13.3)	1.01 (0.99–1.02)
Occiput wall distance (cm)	3.4 (6.0)	3.1 (6.1)	0.99 (0.96–1.02)
Lumbar lateral flexion (cm)	23.7 (19.2)	22.6 (18.8)	0.99 (0.99–1.01)
Hip arthroplasty	9 (2.9%)	5 (2.2%)	0.73 (0.24–2.24)
Inability to work	80 (26.5%)	65 (28.9%)	0.94 (0.63–1.41)

CI: confidence interval; OR: odds ratio. ASDAS: Ankylosing Spondylitis Disease Activity Score; ASDAS-ID: ASDAS inactive disease (ASDAS < 1.3); ASDAS LDA: ASDAS low disease activity (ASDAS < 2.1); BASDAI: Bath Ankylosing Spondylitis Disease Activity Index; CRP (mg/dL): C reactive protein (milligrams/decilitre); SD: standard deviation; VAS: visual analogue scale; SF-12: 12-item Short Form Survey; LDA: low disease activity.

## Data Availability

Data can be shared upon reasonable request and after approval by the scientific committee.
